# Reformative Effects of Intumescent Coating on the Structural Characteristics of Cold-Formed Steel

**DOI:** 10.1021/acsomega.2c06017

**Published:** 2022-11-07

**Authors:** Casim Yazici, Fatih Mehmet Özkal, Suleyman Nazif Orhan, Burak Kaan Cirpici

**Affiliations:** † Department of Construction, Ağrı İbrahim Çeçen University, 04400Ağrı, Turkey; ‡ Department of Civil Engineering, Atatürk University, 25240Erzurum, Turkey; § Department of Civil Engineering, Erzurum Technical University, 25050Erzurum, Turkey

## Abstract

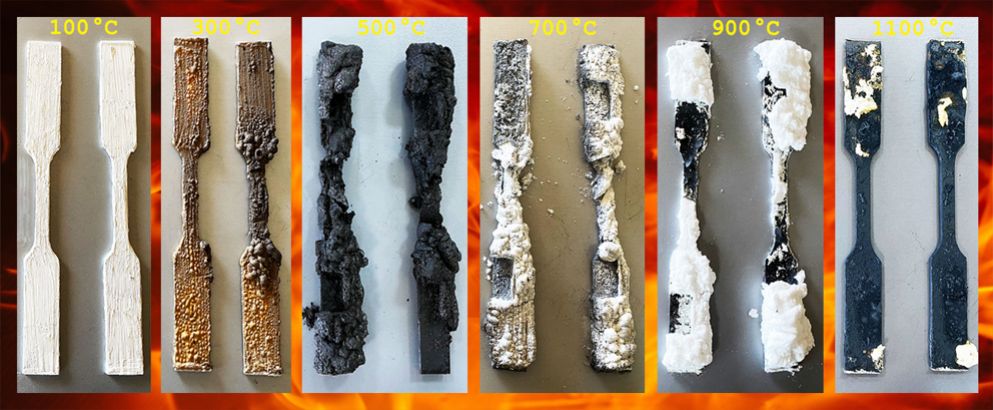

Intumescent fire-resistive coatings are a more recent type of passive fireproofing thin film that swells many times its initial applied thickness, generating an insulating char that functions as a thermal barrier between the fire and structural steel. It keeps the heat of steel members from reaching critical levels and aids in the structural integrity during a fire. They are architects and designers’ favorite choice for passive fire protection of load-bearing steel frame structures because of their aesthetic look, versatility, rapidity of application, and ease of inspection and maintenance. In this study, axial tensile, thermal conductivity, and hardness tests have been performed on S235 cold-formed steel specimens that were exposed to increasing temperature periods. The mechanical behavior of coated and uncoated specimens was investigated over the modulus of elasticity, yield strength/strain, and ultimate strength/strain values for all temperatures. As a result of the research, gradually increasing changes were observed in the mechanical properties of coated and uncoated specimens at increasing temperature levels, compared to each other. However, performance increment on the coated specimens was limited in terms of strength and strain characteristics than expected. Two essential reasons for this conclusion are that the specimens were exposed to heat for a long time after reaching the target temperature and also that the wall thickness of the specimens was thinner with respect to the usual application method of the protective coating. In order to examine the structural properties of the test specimens after elevated temperature effects, thermal conductivity measurement was also performed. Temperature difference between coated and uncoated surfaces provided a benefit in the range of 29–56% due to the coating. Lastly, microstructure imaging techniques demonstrated grain coarsening and no crack development with the increase in temperature.

## 1 Introduction

Thin-walled steel profiles produced by cold-forming method provide mass production and easy installation.^1^ Cold-formed storage rack system members are open sections, and they have indents, protrusions, and holes on their bodies. In recent years, as a result of the increase in distance sales methods, storage rack system demands have continuously been increasing. In line with these increasing demands, storage rack system heights have also been increasing.^2^ Regarding material characteristics, cold-formed steel (CFS) profiles have lower fire resistance compared to hot-rolled profiles. Fire resistance of the material varies depending on the cross-section factor calculated by the relationship between the surface area exposed to fire and the material volume.^3^ Many experimental investigations and finite element analyzes have been carried out in previous studies on the low fire resistance of CFS profiles.^4−8^

Fire resistance and durability of the materials are the outstanding and substantial factors affecting the amount of damage and losses in fires.^9^ While steel structural members have high strength and stiffness under normal conditions, rapid degradation of these characteristics is observed at high temperatures.^10^ Although steel is essentially a non-combustible material with high thermal conductivity, high level of stress that will occur at elevated temperatures or in a fire seriously affects the load-carrying capacity of the structure. An average of 550–600 °C is accepted as the critical temperature range based on the carbon content of steel, and the yield point of steel decreases by more than 50% compared to its initial strength at room temperature.^1^ Strength degradation is encountered alongside the ductility degradation of structural members.^10^ Light steel systems also show great weakness regarding the fire behavior due to the structural characteristics of steel. For this reason, some precautions should be taken in structural fire design. The main objective of the security measures taken against fire is to ensure the safety of life and then to reduce the material damage to the minimum level. These considerations are divided into two main groups as active protection and passive protection techniques.^11^ Active protection is defined as fire safety measures that prevent or delay the spread of fire in buildings, assist in firefighting, and save time for people in the building to escape from the fire. As a general classification, active protection materials are gathered under two basic titles; fire detection/warning systems (detectors and alarm buttons) and fire prevention/extinguishing systems (sprinklers). Passive fire protection systems, on the other hand, are fire protection materials used to delay the temperature increase in the structural members by resisting the fire and delaying the growth, development, and spread of the fire. Passive protection materials are also defined under two titles; fire-reactive and non-fire-reactive materials. Non-reactive protection materials retain their characteristics when exposed to fire at high temperatures and they are most commonly used in coatings and sprays. Reactive protection materials are known with their changeable characteristics with fire, and the most widely used and preferred type is the intumescent paint.^12−16^

Polymeric materials are also used as fire protection material with their low mass and by allowing us to synthesize lightweight structures. However, the apparent disadvantage of these materials is their high flammability.^17^ Intumescent paints (coatings) expand and swell due to the heat and flame they are exposed to during a fire, and they form a foam layer similar to coal by thickening between the flame and underlying substrate.^18^ This foam layer prevents the material surface to contact with air, heat, and fire (barrier effect) and delays the combustion or slows the spread of the fire among the structural system. Depending on the amount of heat that is released in the fire, they can swell up to 2–100 times of their initial thickness and generally provide fire resistance between 30 and 120 min. Intumescent coatings are similar in appearance to conventional paints and are defined as water- or solvent-based (water-miscible), and epoxy-based (mastic or thick-film coatings), which are usually applied with a dry film thickness, no thicker than a few millimeters. They are applied in three layers: a rust-proof primer layer, an intumescent compound layer, and a decorative layer. Intumescent coatings are especially preferred in steel structures because they are significant in terms of architecture and aesthetics and can be applied faster and easier than other passive protective materials, especially on complex surfaces.^19−32^

Thermally reactive intumescent coatings are composed of organic and inorganic components linked together in a polymer matrix.^33−35^ Intumescent coating compositions are typically constituted of an acid source (“catalyst”), a carbonaceous chemical (“carbonific”), a blowing agent (“spumific”), binders, and additives. Their functions have extensively been detailed in previous studies, and formulas have been refined over the last decades to generate an effective protective char. Intumescent process of the coatings can be presented in four steps, basically named as reaction, swelling, char formation, and char degradation.^36^

1 Reaction (melting step): when exposed to a heat source and reaches a threshold temperature, the inorganic acid source undergoes thermal decomposition as the surface melts and transforms into a viscous fluid.^31^
2 Swelling (expanding step): after the melting step, endothermic reactions occur by absorbing heat from the substrate and decomposing it, releasing a large number of gaseous products. Trapped gas bubbles cause the molten matrix to swell up to 2–100 times of its initial thickness depending on the intumescent coating quality, generating a porous medium with low density and thermal conductivity that acts as a thermal barrier for the metal substrate. The swelling process continues until either the blowing ingredient runs out or the carbon matrix becomes too viscous to contain gas bubbles.
3 Char formation (charring step): the porous material hardens and releases residual volatiles to generate char with the increase in temperature. At this stage, the char structure is strongly carbonaceous and has a black-gray color on the exposed surface.
4 Char degradation (change of char structure step): the carbonaceous char oxidizes and CO_2_ releases, gradually transforming the black and compact char structure into a white, brittle, powdery foam at the exposed surface.

The dangers associated with partial or complete structural collapse during or after a fire in a structure can be managed using structural fire safety engineering. In the case of steel constructions, usage of thermal barriers is a common strategy for preventing steel from reaching critical temperatures. This is typically accomplished by coating or wrapping structural components in low density, low thermal conductivity materials that can slow the rate of temperature increase of the load-bearing steel structure.^37^ Historically, a variety of commercially accessible thermal barrier methods that surround steel structures utilizing concrete encasement, calcium silicate or gypsum plaster boards, cementitious spray-on systems, or flexible fire blankets were available. Nonetheless, they have typically been regarded as aesthetically unappealing, and as such, they have not been the most attractive choice for slim and light buildings with exposed steelwork.^38^ As a result, the steel industry has seen a considerable growth in the use of intumescent coatings systems during fire.^21^

The most notable reason for the non-widespread use of fire-retardant paints is their high cost.However, usage of sprinklers, defined as active protection, cannot provide full protection against fire effects because the water released from sprinklers at the ceiling level cannot reach lower shelves and cannot contribute to the extinguishing of fire. As one of the significant studies on fire situations that might occur in storage rack systems, Ren et al.^39^ drew attention to this situation and the structural behavior was investigated by applying the fire effect to the lower level members of the rack system.

The purpose of this study is to investigate to what extent the structural characteristics of rack systems would be preserved after elevated temperature effects if fire-retardant paints, known as passive protection, are applied on CFS. Elevated temperature effects within the range of 23–1100 °C were studied throughout tensile coupon specimens of S235 CFS. Degradation of mechanical properties, hardness, and thermal conductivity characteristics were investigated considering the fire effect on coated and uncoated specimens, alongside optical microscopy and scanning electron microscopy (SEM) image inquisition.

## 2 Experimental Setup

### 2.1 Test Specimens and Coating

**Figure 1:**
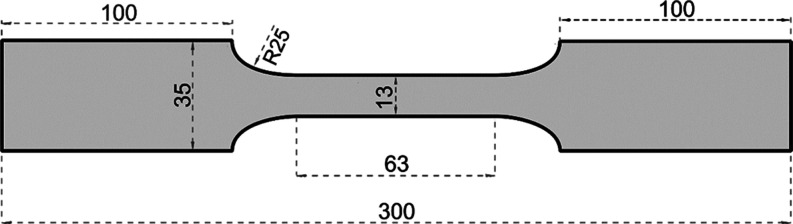
Details of tensile coupon specimens (units in mm).

Tensile coupon specimens were prepared from S235 CFS with a thickness of 3 mm (Figure 1). The specimens were divided into two groups as protected and unprotected against fire. Before the coating process, surfaces of the specimens were thoroughly cleaned and dried. Initially, a suitable coat of anti-rust and priming paint was applied to the surfaces prior to application. The protective coating was mixed to create a homogeneous mixture with an appropriate brush, roller, or sprayer. Following the application of the primer, the paint was applied on the surface in two layers, at 4 h intervals, without diluting, and the painting process was completed. Water-based fire-retardant coating, of which the properties have presented in Table 1, with a thickness of approximately 250 μm was gradually applied to all surfaces of the test specimens. Chemical components of the coating are ammonium polyphosphate (approximately 28%) from ammonium salts as the acid source, pentaerythritol (approximately 10%) for the carbon source as the char former, and melamine (10%) for the blowing agent. The other raw materials are water, cellulose thickener, defoamer, biocide, coalescent, acrylic copolymer emulsion, titanium dioxide, and copolymer dispersion.

**Table 1:** Technical Properties of the Intumescent Coating

ingredient	water-based
color	white (ral colors)
applied temperature	+5∼+35 °C
density	1.20∼1.40 gr/cm^3^
viscosity	10,000∼12,000 mPa·s/25 °C
pH	7.0∼9.0/25 °C
powder drying	45∼60 min/25 °C
touch dry	3 h/25 °C
complete (full) drying	24 h/25 °C

After the coating process was completed, required controls were made with the paint-thickness measuring equipment. A total of 92 specimens were prepared in order to perform four coated and four uncoated tensile tests at target temperature levels.

### 2.2 Exposure to Elevated Temperatures

**Figure 2:**
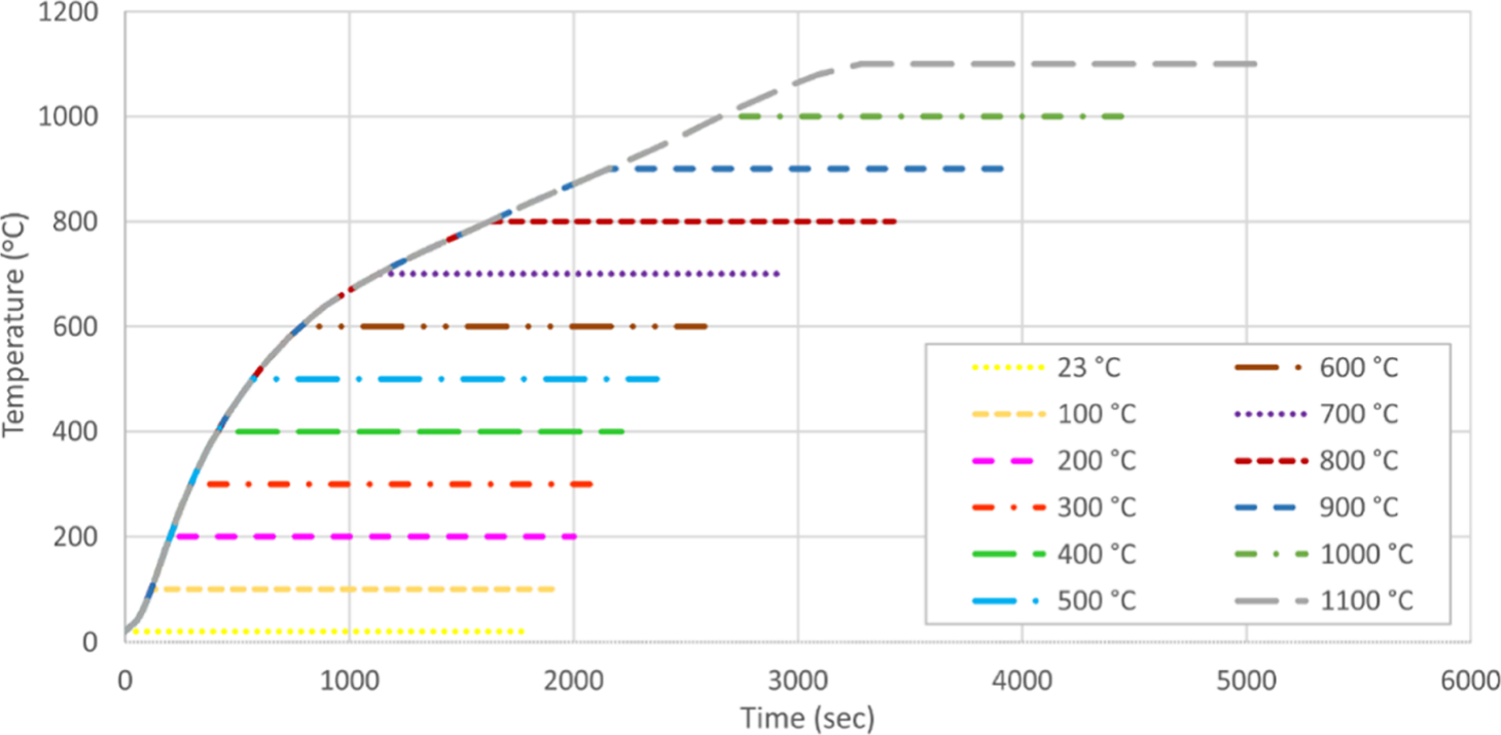
Heating curve of the furnace.

An electric furnace was used for the application of high-temperature effects on the tensile coupon specimens. A K-type thermocouple was placed in the furnace to detect the interior temperature and obtain the heating curve. After waiting for 30 min at target temperatures, tensile coupons were left to cool in the air. Heating curve of the furnace is given in Figure 2.

**Figure 3:**
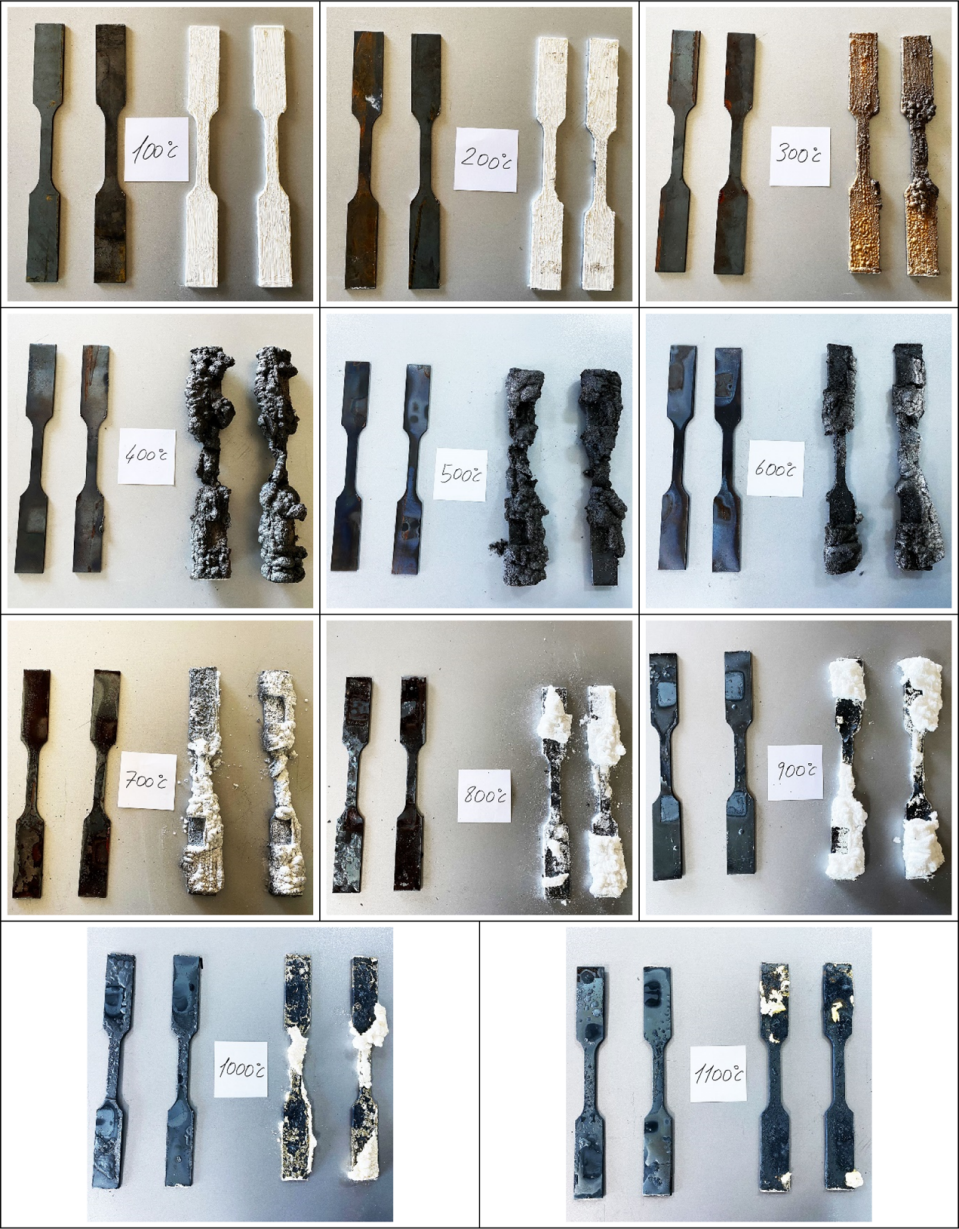
Coated–uncoated test specimens after heating (100–1100 °C).

Test specimens were subjected to axial tensile tests after cooling. Images of the coated and uncoated specimens after heating are presented in Figure 3. The fire-retardant coating started to swell and char at 300 °C and exhibited decomposition and flaking off by taking on a white color again from 700 °C. For uncoated samples, on the other hand, diffraction was observed at the outer layer of the steel from 700 °C. The diffraction depends on the extensive softening of the outer hard layer which generally occurs after 600 °C. This heat level is dependent on the chemical composition of any kind of material and steel has a range of 600–800 °C for outer layer diffraction.^40−42^

## 3 Test Results and Discussion

### 3.1 Tensile Coupon Tests

In order to determine the mechanical properties of coupon specimens, tensile tests were conducted at a rate of 2.5 mm/min in accordance with ASTM E8/E8M-21^43^ regulations. This investigation involved a total of 92 tensile tests (4 for coated and uncoated specimens at their target temperatures). The modulus of elasticity, yield strength/strain, and ultimate strength/strain values of the specimens were determined by analyzing the stress–strain relationship of the test specimens and displaying the results graphically and numerically.

### 3.2 Stress–Strain Relationship

**Figure 4:**
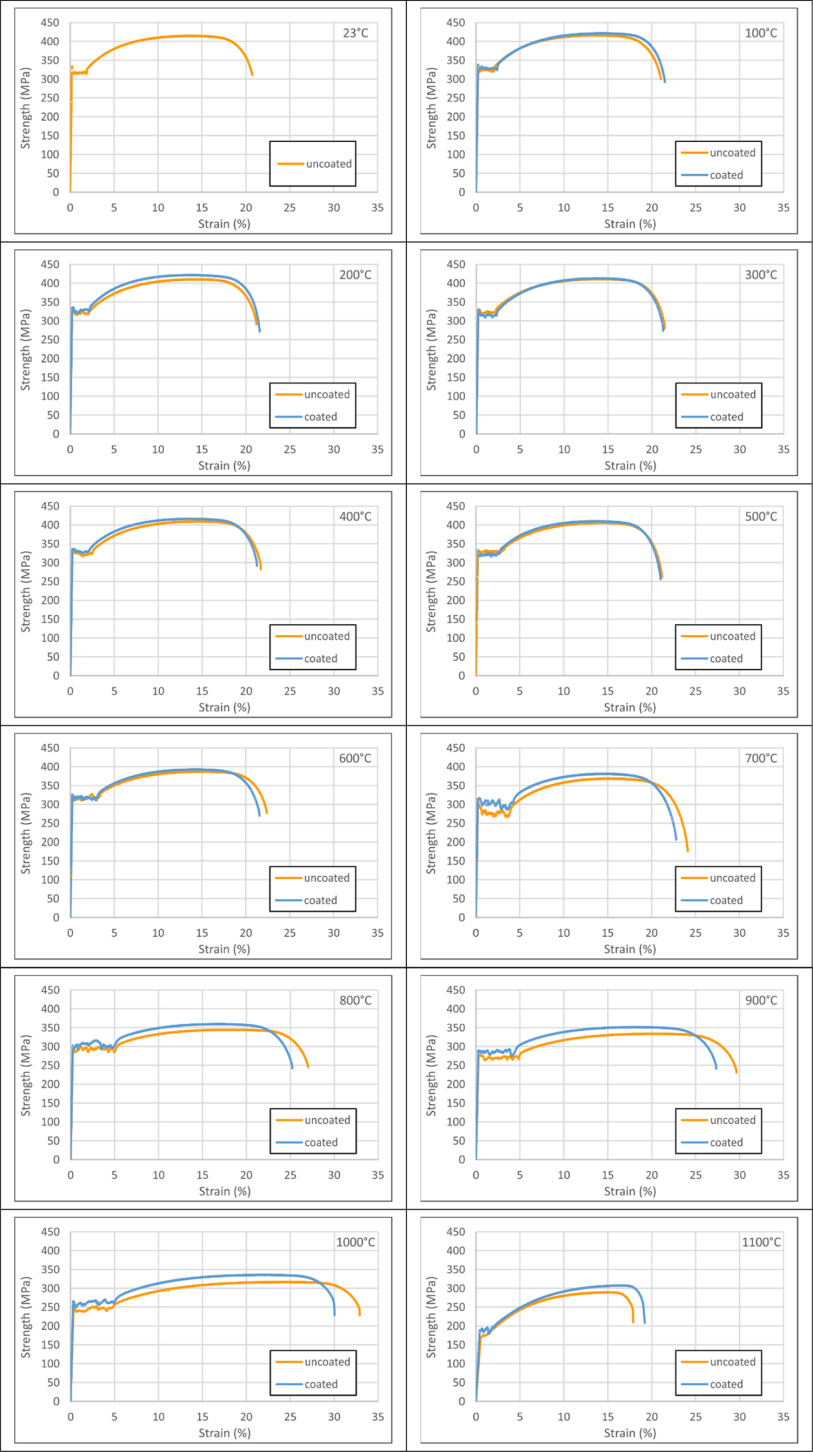
Stress–strain curves of coated–uncoated test specimens (23–1100 °C).

Stress–strain curves of coated and uncoated tensile coupon specimens are demonstrated in Figure 4, indicating that there is not any discernible change in the material behavior up to 600 °C. After this level, increases in yield/ultimate strain and decreases in yield/ultimate strength occur for uncoated specimens, while these changes are more limited for coated specimens. However, the yield zone became uncertain and the ultimate strain value decreased significantly at the 1100 °C heating level. This post-fire behavior has been seen in the studies of Chen et al.,^44^ Lu et al.,^45^ Sajid and Kiran,^46,47^ and Zhou et al.^48^ Post-fire mechanical properties of four commonly used high-strength steel rebar grades (GLG460, GLG550, GLG650, and GLG835) were investigated experimentally by Chen et al.^44^ Specimens were heated to 13 different predetermined temperatures up to 1000 °C and then cooled down to room temperature using two separate methods; air cooling and water cooling. Reduction factor of the yield strength based on air-cooling up to 700 °C is around 1.0, while it is 0.947 for 700 °C and decreasing up to 0.852 for 1000 °C. A similar research with two common structural cast steel (G20Mn5N and G20Mn5QT) has been undertaken by Lu et al.^45^ to investigate the post-heating residual mechanical properties. Residual factors are between 1.0 and 0.981 for the temperature range of 20–700 °C going down up to 0.800 for 1000 °C. Sajid and Kiran^46^ have also mentioned that when specimens were either air-cooled or water-cooled from temperatures up to 600 °C, their post-fire mechanical parameters (yield strength, ultimate tensile strength, and ductility of ASTM A36 steels) remained almost unchanged and decreased by up to 20% when air-cooled from temperatures beyond 600 °C. Regardless of the cooling method, Sajid and Kiran^47^ have also obtained that the post-fire mechanical characteristics of ASTM A572 Gr. 50 steels remained relatively unchanged after exposure to temperatures up to 600 °C. In the study of Zhou et al.,^48^ yield and ultimate strengths of Q620 were unaffected after being exposed to temperatures up to 700 °C with a reduction factor between around 1.0 and 0.9; however, significant changes occurred beyond that with a reduction factor of 0.75–0.71.

### 3.3 Alteration of Material Characteristics

**Figure 5:**
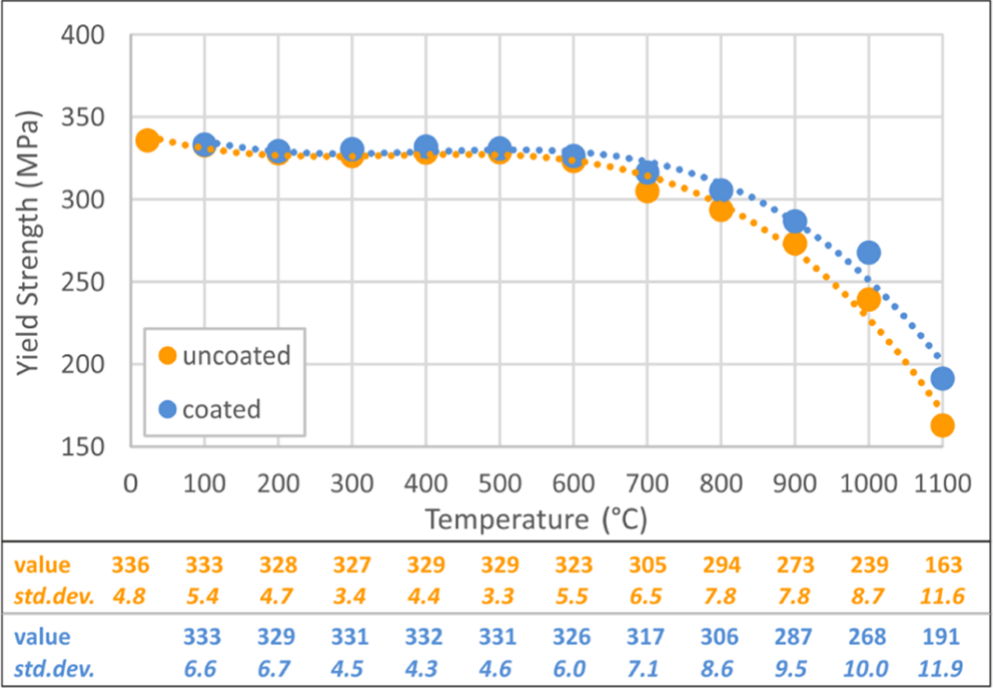
Yield strength values of coated–uncoated specimens.

**Figure 6:**
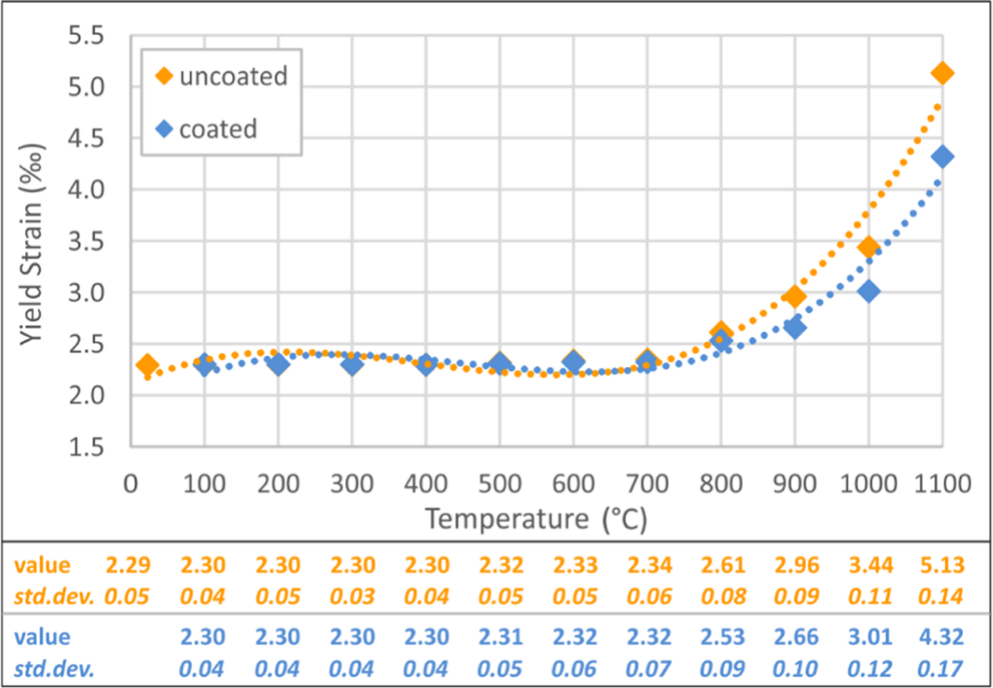
Yield strain values of coated–uncoated specimens.

**Figure 7:**
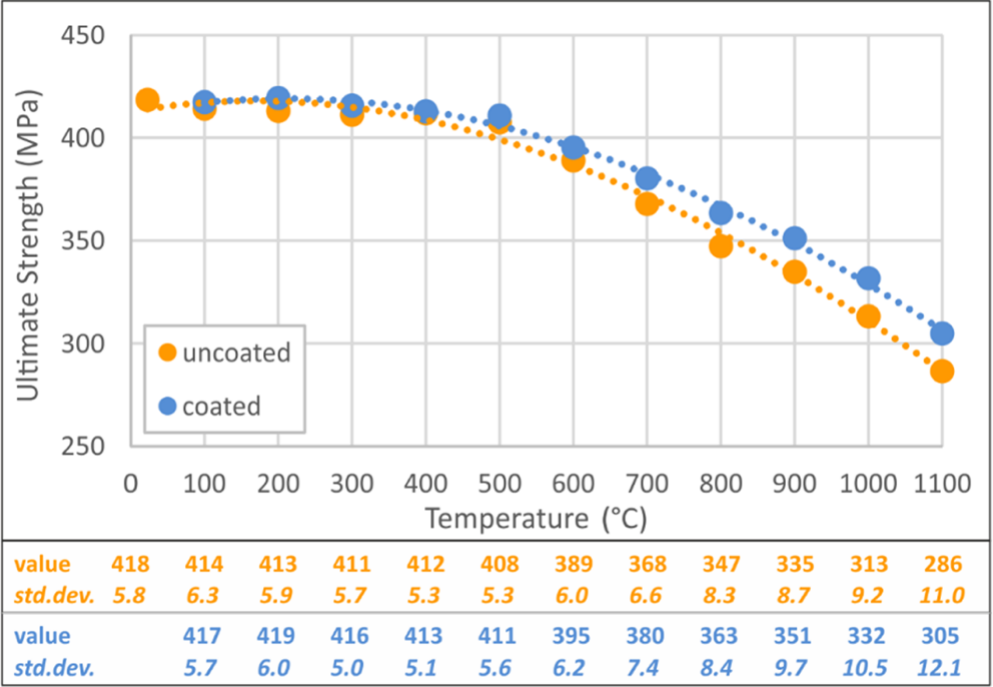
Ultimate strength values of coated–uncoated specimens.

**Figure 8:**
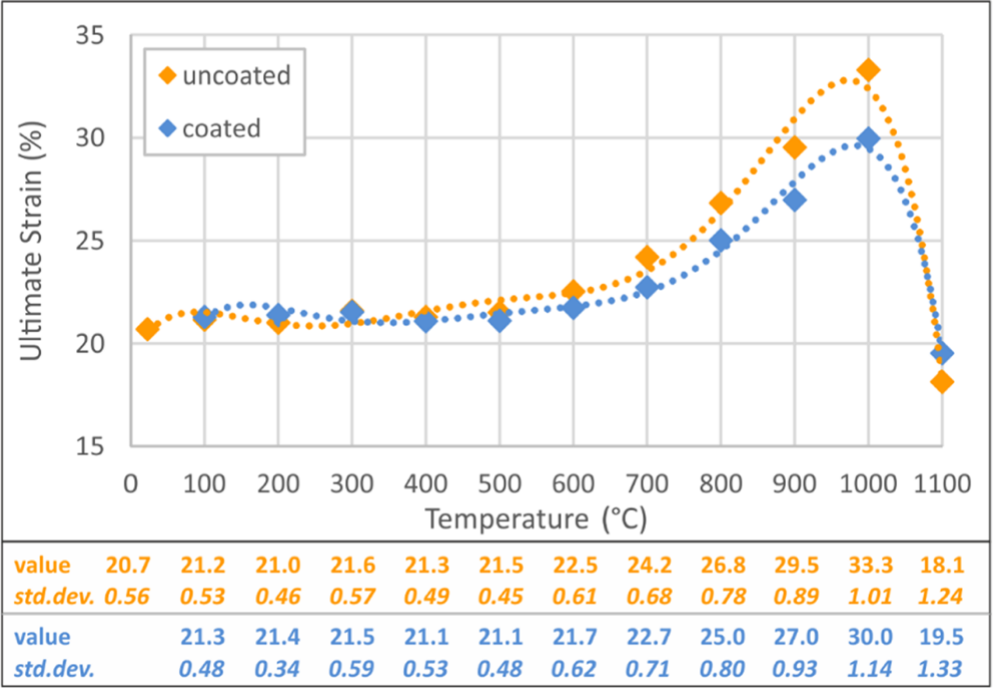
Ultimate strain values of coated–uncoated specimens.

Within the comparison of test results, modulus of elasticity, yield strength/strain, and ultimate strength/strain values were chosen as the mechanical parameters and the proportional values were calculated between the uncoated and coated specimens. Modulus of elasticity was calculated from the slope of the linear part of the stress–strain curve. Yield strength, yield strain, ultimate strength, and ultimate strain values for uncoated and coated specimens are presented in Figures 5–8. These figures reveal that significant changes arise after 600 °C, with an increasing change speed up to 1100 °C. The gap between uncoated and coated specimens increases with the increase in temperature, which proves that protection capacity of the intumescent coating is more effective at higher temperatures.

While there is no remarkable change in general material behavior up to 600 °C, yield and ultimate strength values decrease gradually for all specimens as the temperature increases from this heating level. Due to the performance contribution of the coating protection, there are yield strength and ultimate strength increases of 17.5 and 6.5% between coated and uncoated specimens, respectively. Coated specimens exhibit a decrease in unit strain values of up to 15.8% at the yield state at 1100 °C, a decrease of 10.0% at 1000 °C, and a rise of 7.7% at 1100 °C at the rupture state.

**Figure 9:**
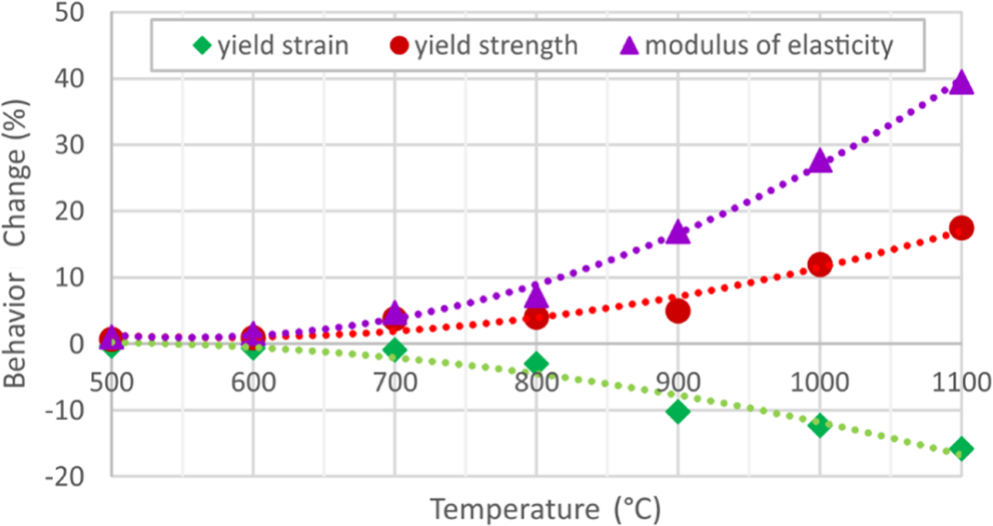
Comparison of yield strength/strain and modulus of elasticity between coated and uncoated specimens.

**Figure 10:**
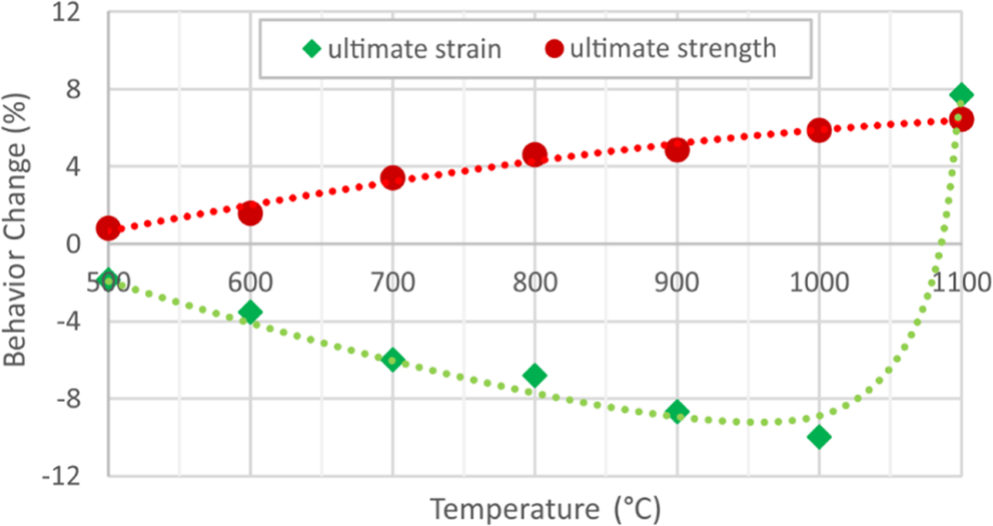
Comparison of ultimate strength/strain between coated and uncoated specimens.

Considering the behavior at 1100 °C, stress–strain curves in Figure 4 and dramatic ultimate strain change in Figure 8 reveal that this temperature level is highly critical regarding the material behavior. Although the intumescent coating has a significant protection capacity, both of the groups (uncoated and coated) achieved an extremely brittle state. The efficiency of the intumescent coating on the modulus of elasticity as percental alteration and also comparisons of strength/strain values for yield/rupture states between coated and uncoated specimens at the range of 500–1100 °C are demonstrated in Figures 9 and 10. The contribution of the intumescent coating on the mechanical characteristics of steel, which improves with the increase in temperature, has also been summarized in Table 2. Temperature increases cause a decrease in the modulus of elasticity and strength values while increasing strain values (except for ultimate strain at 1100 °C). Hence, absolute values for all of the characteristics in these figures and table denote the performance contribution of the coating against elevated temperatures. Coating material keeps steel more rigid and stronger, as evidenced by the preservation of modulus of elasticity by nearly 40% at 1100 °C.

**Table 2:** Contribution of Intumescent Coating on the Mechanical Characteristics of Steel (%)

temperature (°C)	modulus of elasticity	yield strain	yield strength	ultimate strain	ultimate strength
500	1.03	–0.35	0.68	–1.85	0.82
600	1.55	–0.61	0.93	–3.52	1.58
700	4.74	–0.94	3.75	–5.98	3.41
800	7.26	–3.02	4.03	–6.79	4.63
900	16.94	–10.26	4.95	–8.67	4.86
1000	27.69	–12.34	11.94	–9.98	5.88
1100	39.51	–15.80	17.46	+7.72	6.45

### 3.4 Thermal Conductivity Tests

**Figure 11:**
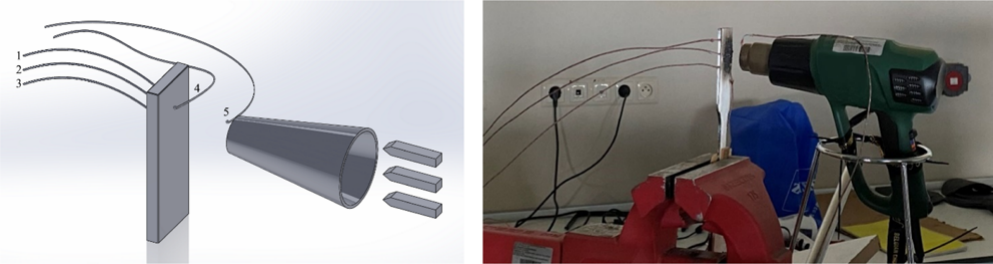
Thermal conductivity test setup.

Thermal conductivity is the intrinsic property of a material that indicates its ability to conduct heat. This property is measured in Watts per meter per degrees Kelvin (W/mK).^49^ Thermal conductivity tests of the intumescent coating were carried out in a Linseis THB-100 measuring instrument at ambient temperature. Schematic test setup for the measurement of coated specimens is shown in Figure 11. Applied thermal load to a single surface has been tried to be characterized by the presence of a thermal barrier coating. The first three thermocouples on the back surface of the coupon record the temperature values on the uncoated surface. The fourth thermocouple records temperature values on the coated surface, and the fifth thermocouple records temperature values on the heater source. The results were evaluated by taking the average value of the three thermocouples on the uncoated back surface.

**Figure 12:**
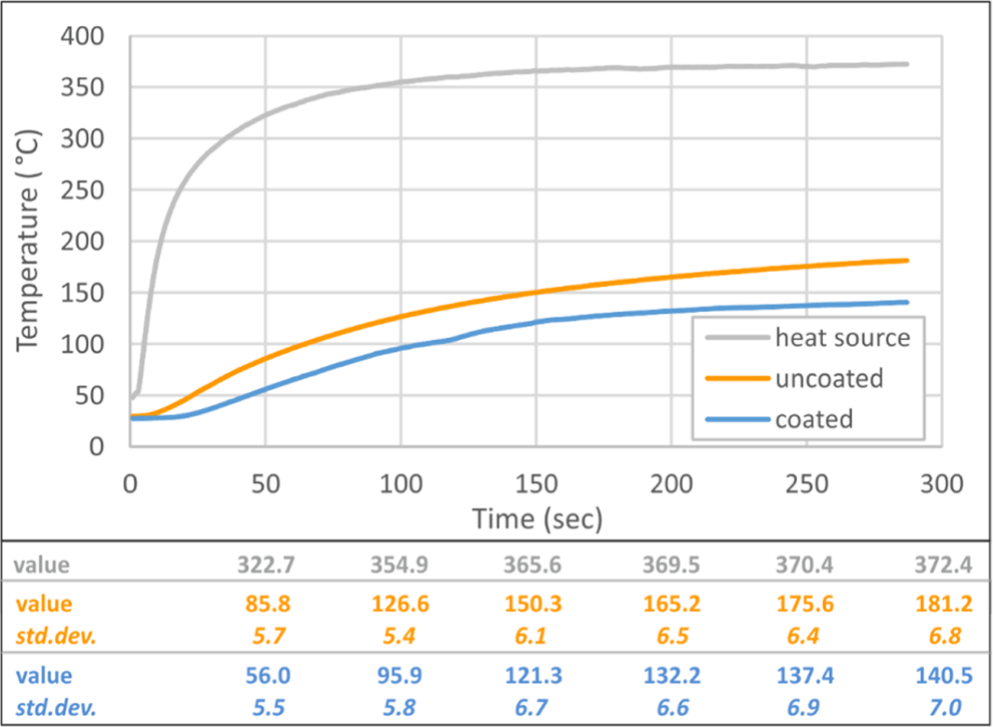
Time-dependent temperature curves of the specimen surfaces.

Thermal conductivity coefficient of the coating was acquired as 0.478 ± 0.013 W/mK at 23 °C. Considering thermal transfer characteristics after coating, it was seen that the coating delays heat transfer. As a result, the coating could not maintain its thermal barrier role until the end of the test, but only extends thermal transition time. Fire protection with the coating caused a delay in the time that the specimens reach target temperature in the homogeneous furnace temperature, but target temperature reached to the whole mass in 30 min. This result was confirmed dependently on the microstructure images and tensile test results. Additionally, it has been observed that intumescent coating protects the material against cross-sectional change as a result of oxidation on the surface. Temperature–time curve of thermal conductivity test is given in Figure 12.

### 3.5 Hardness Tests

Investigation on mechanical characteristics of test specimens after the heating process was also supported by hardness measurements. Because it is simple to perform and does not cause damage on the material, hardness test is one of the most practical mechanical tests. Furthermore, there is a parallel link between the material’s hardness and other mechanical properties, and additional characteristics could be acquired in this manner. Vickers test is the one of the many hardness measurement techniques currently in use. Traditional Vickers hardness tests were carried out at ambient temperature using the Shimadzu GV20 Vickers insert geometry, under 50 g of load, with a waiting time of 15 s. Five measurements from the four specimens of each group were performed, which amounts to 20 measurements for each group and total of 140 measurements from all specimens.

Vickers test uses a four-sided diamond pyramid, of which the side planes are inclined at an angle β = 22° with respect to the specimen surface.^50^ The Vickers hardness commonly used in engineering sciences is defined as load *F* over a superficial impression area *A*_total_
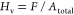
1

Because the side planes are inclined by β = 22°, total area *A*_total_, and projection area *A* are related by *A*_total_ = *A*/cos 22° = 1.08*A*.

**Figure 13:**
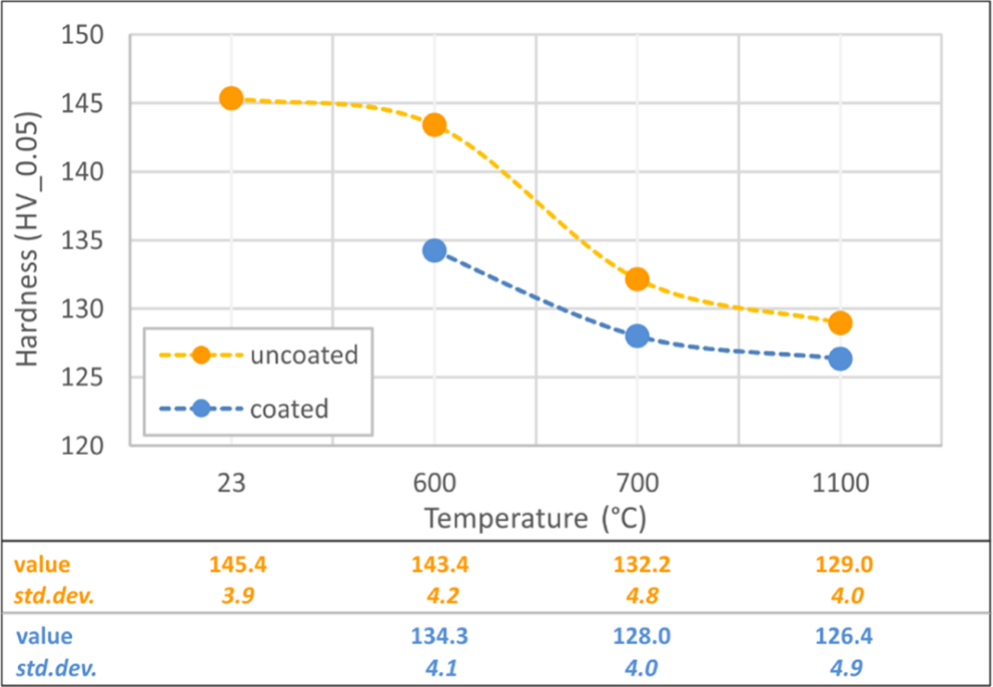
Hardness test results of coated–uncoated specimens.

Hardness test results, which have been achieved at the beginning of the critical heat range (600–700 °C) and the highest level (1100 °C), are provided in Figure 13. The hardness value remains almost constant in its initial state under elevated temperature effects up to 600 °C, but a notable decrease is observed at 700 °C. Although the value decreases at 1100 °C for both uncoated and coated specimens, the degradation is insignificant with respect to the comparison between 600 and 700 °C heating levels. In addition, the overall hardness values of the coated specimens are lower than those of the uncoated specimens, most likely as a result of the interaction between the intumescent coating and the heated surface layer of the steel, which should be discussed over chemical investigations.

### 3.6 Optical Microscope Imaging

**Figure 14:**
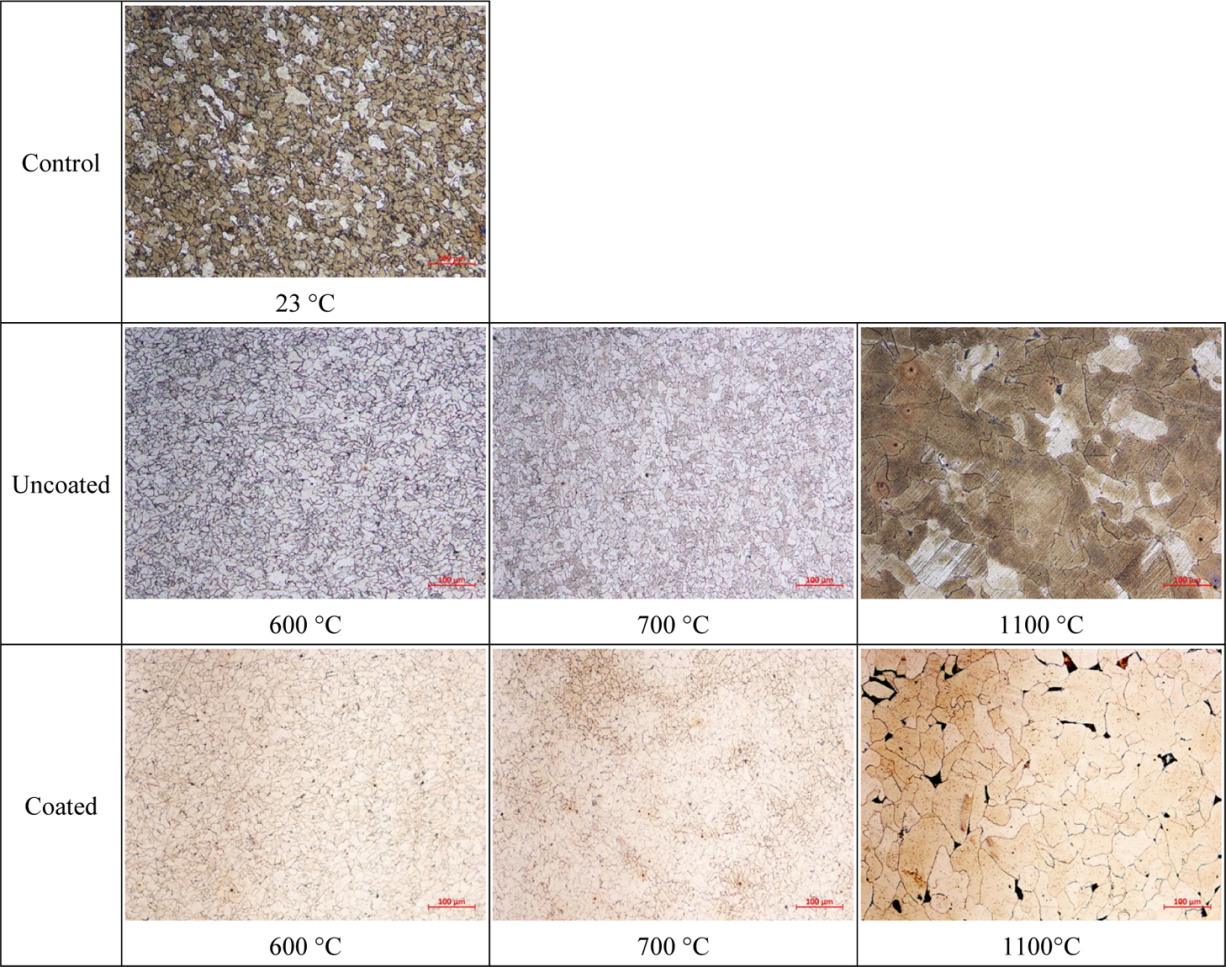
Optical microscopy images of the test specimen surfaces.

Metallographic preparations of the specimens were carried out by polishing the surfaces prepared with 220–1200 level abrasives with 6 μm diamond suspensions and then etching with 5% Nital solution. Optical microstructure images of the coupon surfaces were obtained by the ZEISS AXIO A1 microscope using ZEN software and they are provided in Figure 14.

Similar to the hardness behavior, the microstructure of the specimens exhibits no obvious change at 600 and 700 °C heating levels. However, grain coarsening is seen in the microstructure at 1100 °C owing to the increasing temperature. This obvious variance causes a degradation in the mechanical performance of the specimens, which is also supported by tensile test results. Depending on the applied temperature level and waiting time, it is evaluated that grain coarsening and phase clustering occurs at higher elevated temperatures.

### 3.7 SEM Imaging

**Figure 15:**
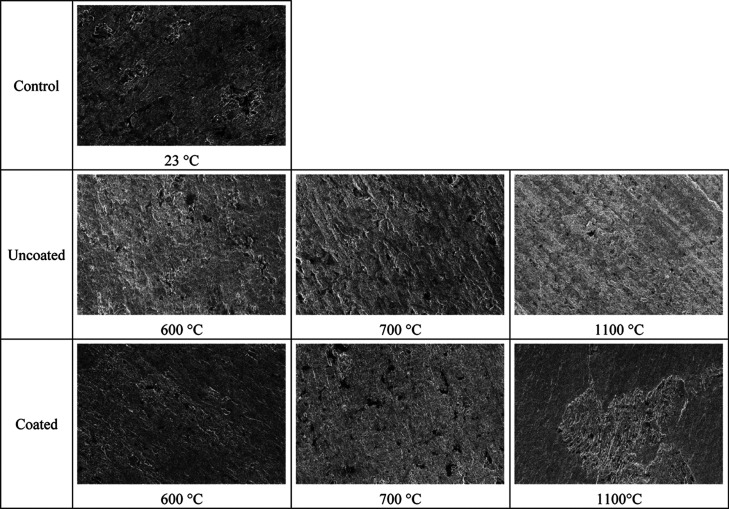
SEM images of the test specimen surfaces.

Using a ZEISS Sigma 300 scanning electron microscope, surfaces of the tensile coupon specimens were examined at a magnification of 2000×. Figure 15 displays SEM images of control, uncoated, and coated samples at various temperatures. Images of specimens depict similar surface appearances, and no cracks were noticed on the surfaces. This is due to the effectiveness of the coating process for fire protection. As is well known, surface fractures can contribute to a reduction in fire resistance as they might negatively affect heat and fire transfer to the steel. Based on the SEM photos, it is possible to conclude that the surfaces have similar appearances and are free of cracks; these findings support the optical microscope images and overall results.

## 4 Conclusions

In this study, axial tensile, thermal conductivity, and hardness tests were performed for the intumescent coated and uncoated states of thin-walled S355 steel profiles produced by the cold-forming method at increasing temperature levels in the range of 23–1100 °C, and following results were acquired. Imaging with an optical microscope and a scanning electron microscope has also been performed to support the research outcomes.

- After elevated temperature effects for coated and uncoated test specimens, almost equal results were obtained in yield and tensile strength values up to 600 °C, while gradual changes were observed in terms of strength and deformation as the temperature increased after 600 °C.
- At 1100 °C, the yield strength suddenly decreased by more than 50%, while the ultimate strength dropped by about 30%. The intumescent coating contributed 17.5% to maintain steel strength in the yield state and 6.5% in the rupture state.
- Yield strain visibly increased from 800 °C up to 1100 °C. Ultimate strain exhibited divergency at 1100 °C and the material behavior became brittle by showing even lower strain than the control specimen. With intumescent coating, these changes are limited to 15.8% in yield and 7.7% in rupture states.
- Decrease in the modulus of elasticity values at 1100 °C was 78.3% for uncoated specimens and 69.7% for coated specimens. Contribution of the coating to the preservation of the modulus of elasticity was observed as 39.5%.
- According to the measurements of thermal conductivity, the temperature difference between coated and untreated surfaces ranges from 29 to 56%, which is regarded as a substantial heat preservation by the coating.
- The hardness degradation appeared to begin between 600 and 700 °C, with an 11.3% degradation ratio for uncoated specimens. However, steel-coating interaction yielded less hardness compared to uncoated specimens.
- Up to 600 °C, optical microscopy and SEM images revealed no obvious change in the microstructure. However, grain coarsening and phase clustering occur at 1100 °C without the formation of cracks.
- Although intumescent coating could be expected to exhibit more efficiency in the preservation of the structural properties, two main reasons are in evidence. At first, exposing the test specimens to the target temperature in the furnace for 30 min limited the efficiency of the coating material. Considering the thinner thickness of tensile coupons (3 mm) compared to thick-walled structural members, protection of the intumescent coating remained at a lower level than its actual capacity.
